# Epidemiology, Management, Quality of Testing and Cost of Syphilis in Germany: A Retrospective Model Analysis

**DOI:** 10.3389/fpubh.2022.883564

**Published:** 2022-04-26

**Authors:** Renata Šmit, Nathalie Wojtalewicz, Laura Vierbaum, Farzin Nourbakhsh, Ingo Schellenberg, Klaus-Peter Hunfeld, Benedikt Lohr

**Affiliations:** ^1^Northwest Medical Centre, Medical Faculty, Academic Teaching Hospital, Institute for Laboratory Medicine, Microbiology and Infection Control, Goethe University, Frankfurt, Germany; ^2^INSTAND e.V. Gesellschaft zur Foerderung der Qualitaetssicherung in Medizinischen Laboratorien e.V, Duesseldorf, Germany; ^3^Center of Life Sciences, Institute of Bioanalytical Sciences (IBAS), Anhalt University of Applied Sciences, Bernburg, Germany

**Keywords:** syphilis, healthcare utilization database, blood donor database, Germany, retrospective model analysis, EQA, economic model

## Abstract

**Background:**

A multi-dimensional model can be a useful tool for estimating the general impact of disease on the different sectors of the healthcare system. We chose the sexually transmitted disease syphilis for our model due to the good quality of reported data in Germany.

**Methods:**

The model included gender- and age-stratified incident cases of syphilis (in- and outpatients) provided by a German statutory health insurance company, as well as seroprevalence data on syphilis in first-time blood donors. Age standardized rates were calculated based on the standard German population. The test quality was assessed by extrapolating the number of false-positive and false-negative results based on data from Europe-wide external quality assessment (EQA) schemes. The model analysis was validated with the reported cases and diagnosis-related group (DRG)-statistics from 2010 to 2012. The annual direct and indirect economic burden was estimated based on the outcomes of our model.

**Results:**

The standardized results were slightly higher than the results reported between 2010 and 2012. This could be due to an underassessment of cases in Germany or due to limitations of the dataset. The number of estimated inpatients was predicted with an accuracy of 89.8 %. Results from EQA schemes indicated an average sensitivity of 92.8 % and an average specificity of 99.9 % for the recommended sequential testing for syphilis. Based on our model, we estimated a total average minimal annual burden of €20,292,110 for syphilis on the German healthcare system between 2010 and 2012.

**Conclusions:**

The linking of claims data, results from EQA schemes, and blood donor surveillance can be a useful tool for assessing the burden of disease on the healthcare system. It can help raise awareness in populations potentially at risk for infectious diseases, demonstrate the need to educate potential risk groups, and may help with predictive cost calculations and planning.

## Introduction

Syphilis is a systemic disease caused by the bacteria *Treponema pallidum*. The pathogen can be transmitted via transplacental transmission, sexual contact with infectious lesions, and blood transfusions (1). Untreated or undetected infections can lead to severe health outcomes (f. e. neurosyphilis) and can even compromise pregnancy outcomes (including stillbirth and congenital syphilis) (2); thus it represents a serious health concern.

While treatment of syphilis is assessable and cost effective, the diagnosis of syphilis is challenging because traditional tools like cultivation and gram staining are not available (3). In addition, the clinical symptoms often indicate more than one possible differential diagnosis result (4, 5). Since the disease tends to manifest inconspicuously, an infection often remains undetected (6), resulting in an underassessment of infections. Currently, most syphilis cases are diagnosed through serological testing (7) and, in Germany, all laboratories are obliged to anonymously report treponemal pallidum positive serological test results to the Robert Koch Institute (RKI) (8). This offers a good insight into incident cases. Syphilis antibodies detected in blood donor samples must also be reported to the RKI (9). Transmission via transfusion has not happened for over 15 years in Germany (10). However, blood donor data is a useful tool in providing information on the seroprevalence of syphilis in the population, making it suitable for monitoring the effects of public health programs (11, 12).

Nevertheless, these sources offer only limited information on some aspects, like treatment patterns and loss of productivity due to sick leave. Claims data from statutory health insurance companies can close this information gap since they reflect real-life healthcare provisions better than clinical trials (13).

Combining different datasets into a model analysis is a helpful tool for developing recommendations and guidelines and for initiating effective public health measures (13).

The aim of this study is to provide a robust, multi-dimensional model analysis of the possible impact of syphilis on the German healthcare system in order to support healthcare decision-making by linking various health-related data sources.

It is the first study to combine cross-validated data from a German statutory health insurance company with information on seroprevalence derived from blood donor screening data from 2010 to 2012 and to conduct an evaluation based on actual data and diagnosis-related group (DRG) statistics, reported during this period. The claims data and blood donor data are normalized to the German population in the observed period of time as a retrospective model analysis to estimate the diagnostic and economic burden on the German healthcare system. The normalization of both datasets to population levels should act as an indicator of any underestimation as a result of either underreporting or underassessment of syphilis cases in Germany. Furthermore, data from Europe-wide external quality assessment (EQA) schemes are used to access the current data on the quality of *in vitro* serological testing of *Treponema pallidum* and its impact on the German healthcare system. These EQA schemes are conducted by INSTAND, one of the three organizations in Germany designated as a reference institution by the German Medical Association.

## Materials and Methods

### Analysis of Health Insurance Datasets

The basic dataset consisted of health insurance data from the German statutory health insurance company *Deutsche Angestellten Krankenkasse-Gesundheit* (DAK-G) from 2010 to 2012 and covered around 5.8 million people insured during the study period. The relevant international classification of disease (ICD-10-German Modification) for syphilis was used: syphilis (A50. - congenital syphilis, A51.x - early syphilis, A52.x - late syphilis, A53.x - other or unspecified syphilis). Data were available up to December 31, 2012 (Supplementary Tables 1, 2). All analyses were based on anonymized subject-specific data. The personal data were exclusively handled by DAK- G in accordance with legal data protection requirements. Information on comorbidities was not included in this model, since we wanted to focus on the sole impact of syphilis. The quality of the data was checked for completeness, correct usage of inclusion criteria, and plausibility prior to analysis according to existing standards (14, 15). Incident cases of syphilis diagnosed on an inpatient and outpatient basis in 2010, 2011, and 2012 were analyzed and extrapolated to the German population. Incident cases were defined as follows: diagnostic code A50.x, A51.x, A52.x or A53.x, identifier “G” indicating a confirmed diagnosis (16) and the concurrent treatment with a suitable antibiotic (J01CE08, J01AA02, J01DD04) in the corresponding quarter of the year. Informed consent is not required for these analyses in Germany.

We extracted patient data (subject specifier, gender, year of birth, code for current residence, date of begin and end of insurance) and treatment procedures (inpatient, outpatient, medication). Additionally, data on productivity loss were included to assess possible indirect costs using the human capital method. Reported sick leave time of inpatients right before or after the hospitalization was attributed to the inpatient cohort.

### Epidemiological Data

The reported *Treponema pallidum*-positive lab results are accessible in a simplified form via the German database SurvStat@RKI 2.0. The number of syphilis cases for 2010, 2011, and 2012 was retrieved from the database by age (5-year interval), gender and region (17). Residual titers of past infections, suspected double reporting, as well as suspected cases of insufficiently treated syphilis (*syphilis non satis curate*) were excluded from this dataset by RKI prior to the analysis (18).

The seroprevalence of syphilis in blood donors was kindly provided by RKI and is based on blood donor surveillance data (9). The number of positive blood donors was calculated based on the reported seroprevalence rates and the corresponding number of blood samples. We only analyzed data on new blood donors as repeat blood donors are repeatedly screened. Thus they are less likely to be infected and are considered a low-risk population for blood-borne diseases (19). It should be noted that the statistical power of this small study population would be insufficient for further extrapolations.

### German EQA Schemes for *Treponema* Pallidum

Between 2010 and 2012, six EQA surveys for syphilis were conducted by the German Society for Promoting Quality Assurance in Medical Laboratories (INSTAND) in cooperation with the central reference laboratory at the Institute for Laboratory Medicine, Microbiology & Infection Control at the Northwest Medical Center, Frankfurt/Main (Germany) and with the six reference laboratories of the Bacteriologic Infection Serology Study Group of Germany (BISSGG). Previous reports summarize the organization, structure and detailed evaluation procedures of the German EQA program for bacteriologic infection serology (20–22).

Participants can report qualitative and quantitative results together with additional information on the test kit provider, lot number and laboratory equipment used. In this study, the accuracy of the qualitative as well as the quantitative results were evaluated for TPPA, TPHA, VDRL, FTA-abs IgG and FTA-abs IgM. All EQA samples are derived either from patients with a confirmed diagnosis of syphilis or from healthy blood donors, where absence of *Treponema pallidum* antibodies was confirmed prior to the EQA survey.

### Statistical Analysis

The data on the insured individuals and the epidemiological data were stratified by gender, age (<25, 25–34, 35–44, 45–54 and >54) and, in the case of the health insurance dataset, 5-digit postal codes. The data were standardized to the general population of Germany for the corresponding years. German population data for 2010, 2011, and 2012 were obtained from the official reports published by the Federal Statistical Office (www.destatis.de). Age-distributed annual incidences were calculated. For the model analysis, age standardized incidence rates (ASR) from the health insurance dataset and age standardized seroprevalence rates from the blood donor dataset were calculated for 2010, 2011, and 2012 to allow comparisons to be drawn with the incidence of reported cases. 95 %-confidence intervals were calculated based on the assumption of a Poisson distribution of the reported cases (23). Congenital syphilis was excluded from the standardization of the population level due to the low numbers and the thorough screening system for pregnant women in Germany.

To calculate the averages for sensitivity, specificity and accuracy (pass rate) from the German EQA survey data (Supplementary Table 3), the reported diagnoses of sample donors were used as the “gold standard”. Average net sensitivity and average net specificity were calculated by sequential testing (two-stage screening) using TPHA/TPPA or ELISA as the first test and FTA-abs IgG, IgG Blot, IgG ELISA methods or TPHA/TPPA as the second test. This test algorithm is currently recommended in Germany (16, 24). Average sensitivity and average specificity were used to calculate false positives, false negatives, true positives, and true negatives based on the standardized incidence or, in the case of blood donors, the seroprevalence. Positive predictive values (PPV) and negative predictive values (NPV) were calculated using Bayes' theorem (25).

### Cost Analysis

For this study, we calculated direct medical costs for inpatient and outpatient treatment, screening, and confirmatory testing, as well as indirect costs from loss of productivity for 1 year. Indirect costs from loss of productivity were calculated using existing German standards (Hanover consent). Our estimates of indirect costs were based on average earnings (€ 3,014) (26) and the median number of productivity days lost for German syphilis patients aged between 18 and 64. Short-term (<3 days) absence from work without a doctor's note was not included due to lack of data. The serological testing costs for statutory health insurance patients (~ 90 % of the German population) and the blood donor population were calculated using the diagnostic claims code “*Einheitlicher Bewertungsmasssta*b” (EBM) (27), while the costs for the serological tests for the privately insured patients (~10 % of the population) were calculated based on “*Gebuehrenordnung fuer Aerzte”* (GoAe) (28). The percentage of antibiotics prescribed in our insurance dataset were used to calculate medication costs (Supplementary Table 4). We were unable to calculate treatment costs for patients coded with unspecified syphilis (A53.x), since we had no information about the detailed dosage and duration of therapy. Therefore, these patients were excluded from the cost analysis. Total costs calculated from the claims data were extrapolated to the German population.

## Results

### Summary of Reported Cases of Syphilis From all Datasets

Table 1 shows the general distribution of syphilis cases in all datasets, including basic characteristics of the corresponding populations. The average ages of the DAK-G cohort and the German population were comparable, while the first-time blood donor cohort was notably younger. The insurance dataset consisted of a higher proportion of women to men than the other two datasets.

**Table 1:** Basic characteristics of the datasets used for this model analysis for 2010 to 2012.

	**2010**	**2011**	**2012**	**Average**
^(a)^				
Population	81,751,602	80,327,900	80,523,746	80,867,749
Male to female ratio	1:1.0	1:1.1	1:1.1	1:1.0
Average age men	42	42	42	42
Average age women	44	45	45	45
No. of syphilis cases	4,077	4,633	5,012	4,574
Incidence rate / 100,000 person-years	5.0	5.8	6.2	5.7
Male to female ratio	10.0:1	11.8:1	12.5:1	11.5:1
Average age cases men	40	40	40	39
Average age cases women	39	39	37	39
	**2010**	**2011**	**2012**	**Average**
^(b)^				
Population	6,119,470	5,800,795	5,683,710	5,867,922
Male to female ratio	1:1.5	1:1.5	1:1.5	1:1.5
Average age men	41	41	41	41
Average age women	48	48	48	48
No. of syphilis cases	438	359	317	371
Incidence rate / 100,000 person-years	7.2	6.2	5.6	6.3
Male to female ratio	2.7:1	3.3:1	3.1:1	3.2:1
Average age cases men	48	47	48	48
Average age cases women	66	62	65	65
	**2010**	**2011**	**2012**	**Average**
^(c)^				
No. of samples from first-time donors	561,642	542,492	496,771	533,635
Male to female ratio	n.I.	1:1.0	1:1.0	1:1.0
Average age men	n.I.	n.I.	n.I.	26
Average age women	n.I.	n.I.	n.I.	26
No. of anti-Treponema positive samples	236	223	221	227
Seroprevalence / 100,000 blood samples	42.1	41.1	44.4	42.5
Male to female ratio	2.2:1	1.7:1	2.1:1	1.9:1
Average age cases men	1 37	37	37	37
Average age cases women	1 43	41	44	43

(a) *Reported data on total German population ( www.destatis.de) and reported syphilis cases (RKI) (17).*

(b) *DAK-G dataset.*

(c) *First-time blood donors (blood donor surveillance, hosted by the RKI) (12, 29). Missing data was coded n.I. (no information)*.

**Figure 1:**
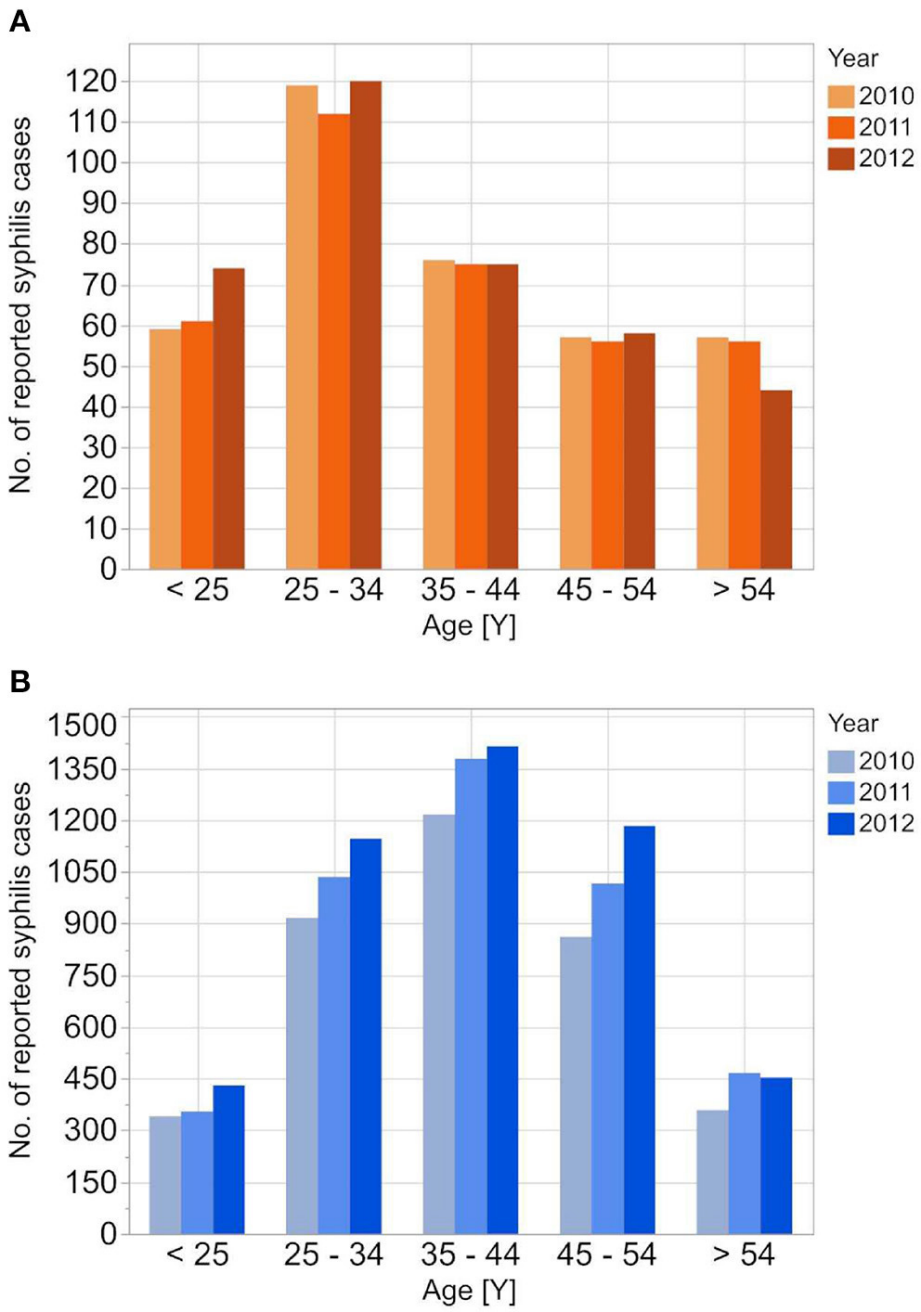
Age stratified reported incidences of syphilis reported to RKI from 2010 to 2012 for the female **(A)** and the male **(B)** population.

**Figure 2:**
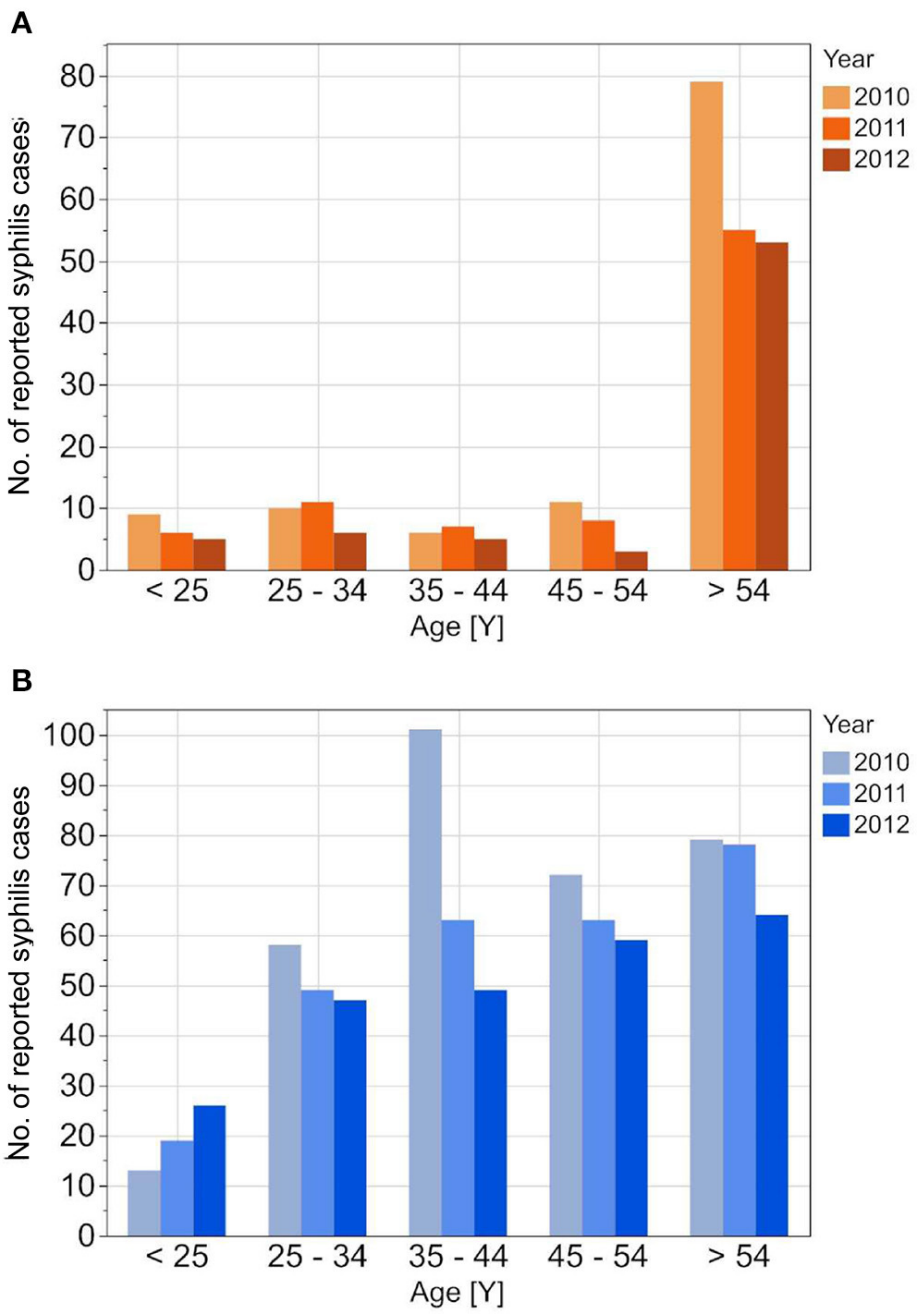
Age stratified reported incidences of syphilis in DAK-G insurance data set from 2010 to 2012 for the female **(A)** and the male **(B)** insured population.

**Figure 3:**
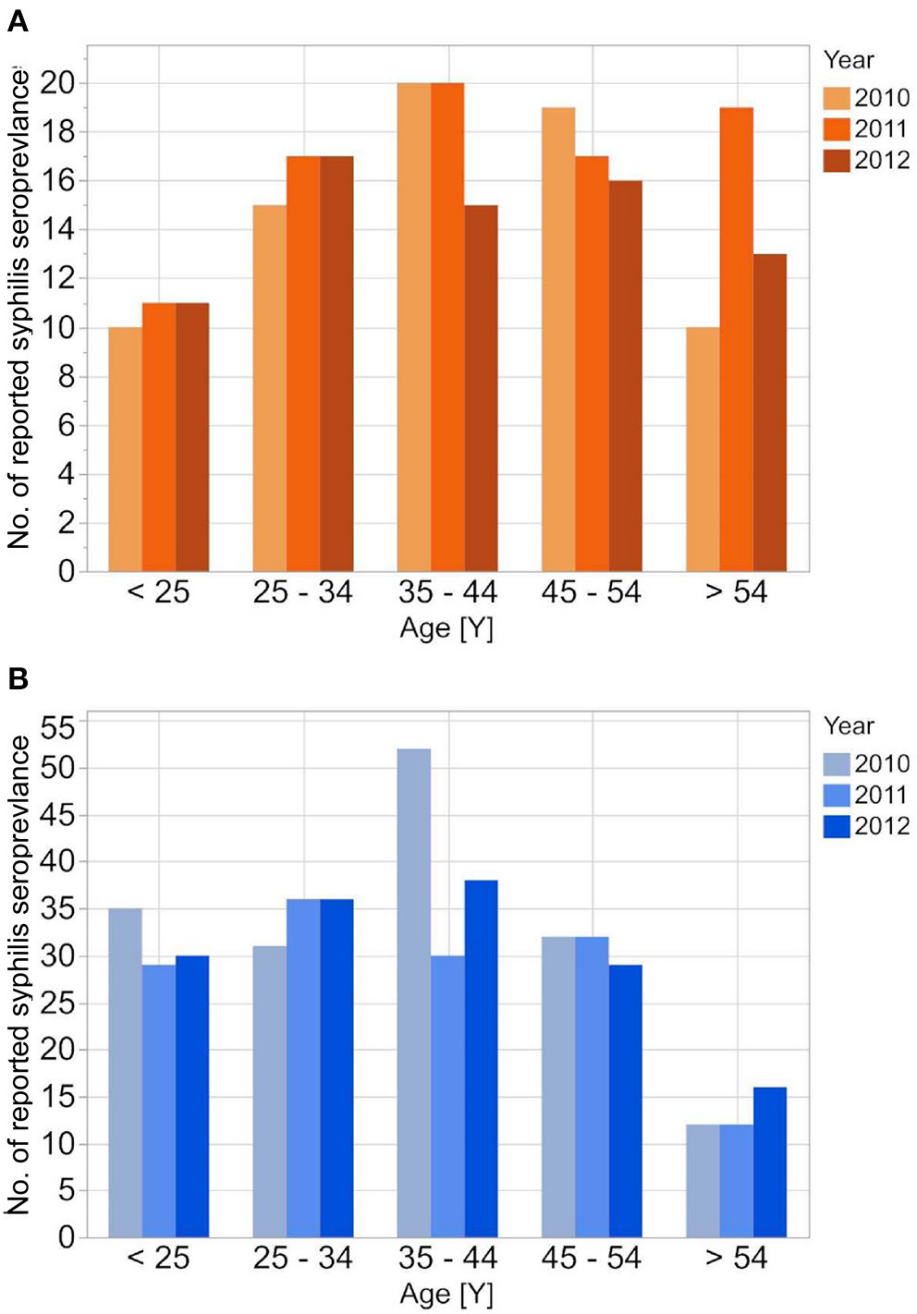
Age stratified reported seroprevalence of syphilis in first time blood donors from 2010 to 2012 for female **(A)** and male **(B)** donors.

The RKI data showed higher male-to-female ratios in the reported incident syphilis cases than the other two datasets, while the average age of syphilis patients was highest in the DAK-G dataset. The age-stratified distribution of the syphilis cases from the individual datasets are presented in Figures 1–3.

The reported cases show the highest number of cases for men in the age group 35–44 years; the highest number of cases for women is between 25 and 34 years. In contrast to the reported cases, the number of incident cases in the DAK-G dataset was highest in the older age groups, especially for women, where most cases were observed in the age group > 54 years. The largest proportion of all seropositive new donors was seen in the age group 35–44 for both sexes.

The insurance cohort included more detailed information about the diagnosis as well as the form of therapy. Of all incident diagnoses, 87 % were outpatients and 13 % inpatients. Most cases were coded with either early syphilis (33 %), late syphilis (34 %) or other/unspecified syphilis (30 %), while 3 % of the incident diagnoses were coded congenital syphilis.

### Extrapolation to Population Level

The syphilis incidence of the insurance and the donor datasets was extrapolated to the population level by calculation of age standardized rates (ASR), since the age structure of both study populations differs from that of the reference population.

The ASR for syphilis incidence based on the insured population dropped from 7.4 [CI: 6.7–8.1] incident cases per 100,000 standardized person-years in 2010 to 5.9 [CI: 5.2–6.5] incident cases per 100,000 standardized person-years in 2012.

**Figure 4:**
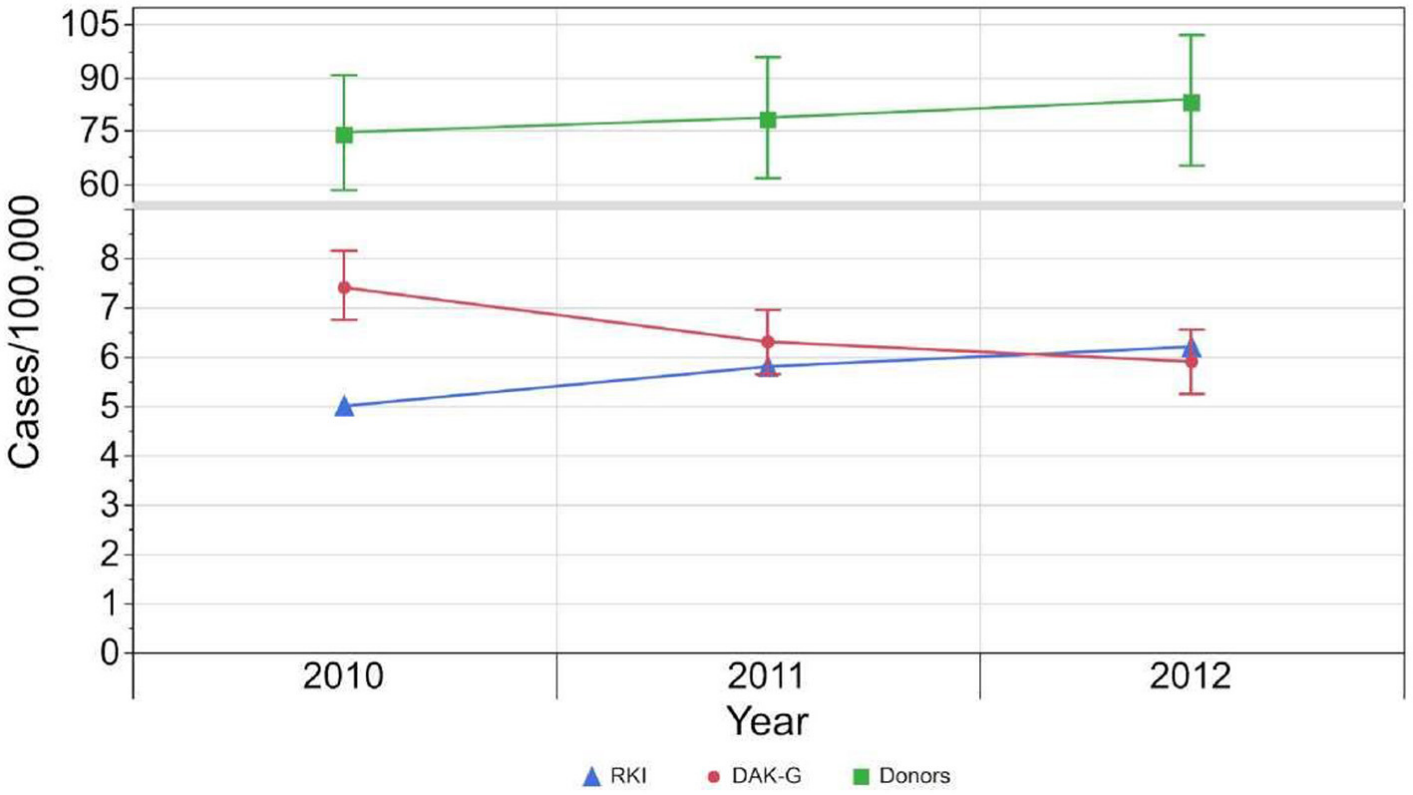
Development of incident cases reported to RKI (blue), standardized incident cases based on health insurance data set (red) and standardized seroprevalence (green) per 100,000 standardized population from 2010 to 2012. The 95 %-confidence intervals of the estimated results per 100,000 standardized person-years are indicated by the whiskers.

In the case of first-time donors, the estimated seroprevalence rate rose from 73.7 [CI: 57.4–89.9] cases per 100,000 standardized person-years to 82.9 [CI: 64.5 - 101.3] cases per 100,000 standardized person-years (Table 2, Figure 4).

**Table 2:** ASR for the insurance data set and the first-time blood donor cohort from 2010 to 2012.

**Dataset**	**Year**	**ASR/100.000 person-years**	**95 %-CI**
DAK-G	2010	7.4	6.7 - 8.1
	2011	6.3	5.6 - 6.9
	2012	5.9	5.2 - 6.5
First-time blood donors	2010	73.7	57.4 - 89.9
	2011	78.0	60.8 - 95.2
	2012	82.9	64.5 - 101.3

### Geographical Distribution of Estimated Cases

**Figure 5:**
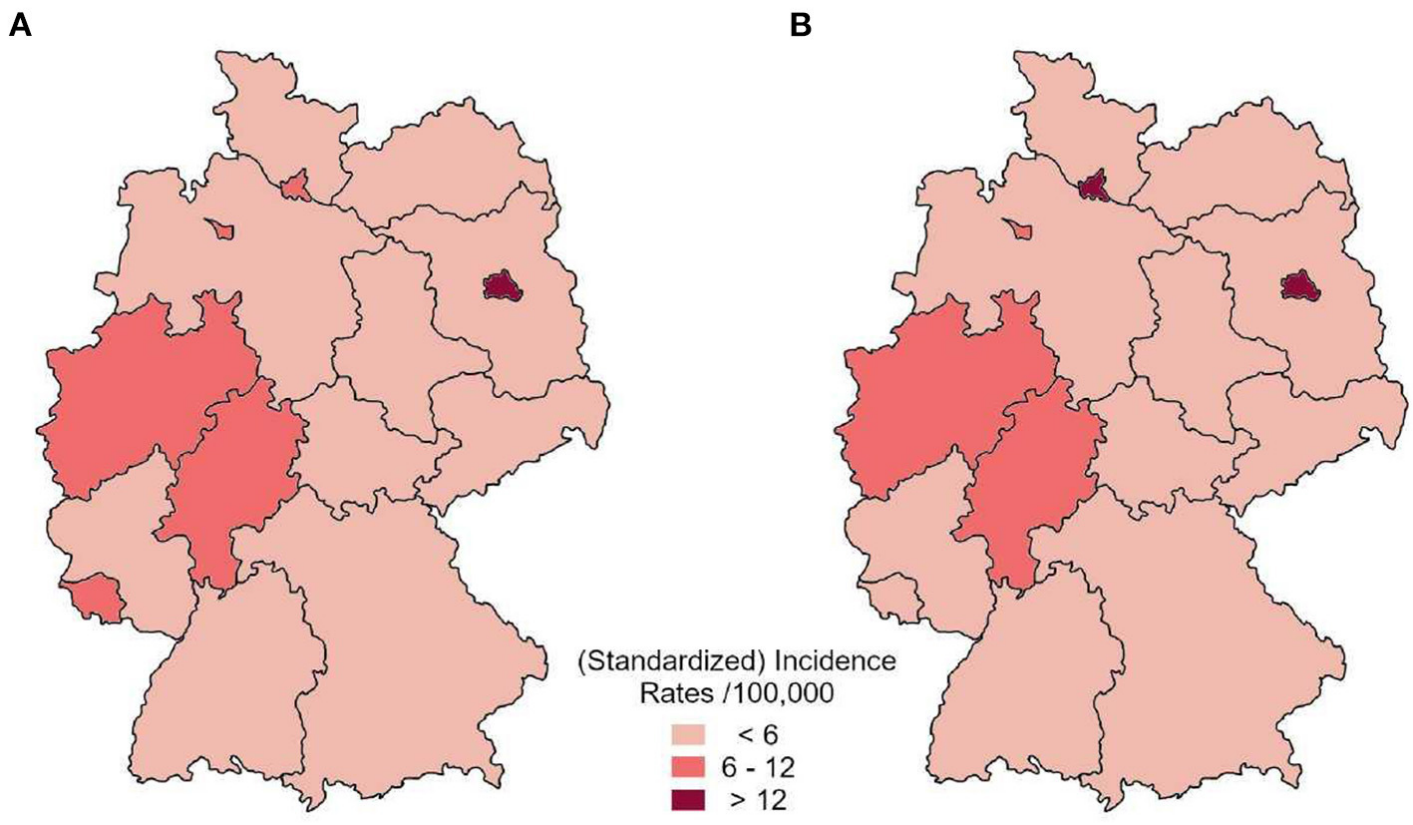
Distribution of average annual standardized incidence by federal state based on the health insurance dataset **(A)** in comparison to reported incident cases to RKI **(B)** between 2010 and 2012.

Since the insurance dataset contained the patient's current postal code, it was possible to extrapolate a geographical distribution of the expected syphilis cases based on the extrapolated and standardized insurance data (Figure 5A).

The highest standardized incidence was estimated for Berlin (> 12 expected cases per 100,000 standardized person-years). Hamburg, Bremen, North Rhine-Westphalia, Hesse and Rhineland-Palatinate showed between 6 and 12 expected cases per 100,000 standardized person-years. All other federal states showed a lower estimated incidence rate. This distribution is in line with the Germany-wide pattern of the actual incidence cases reported to RKI (Figure 5B).

### Results From Germany's Proficiency Testing Program

**Figure 6:**
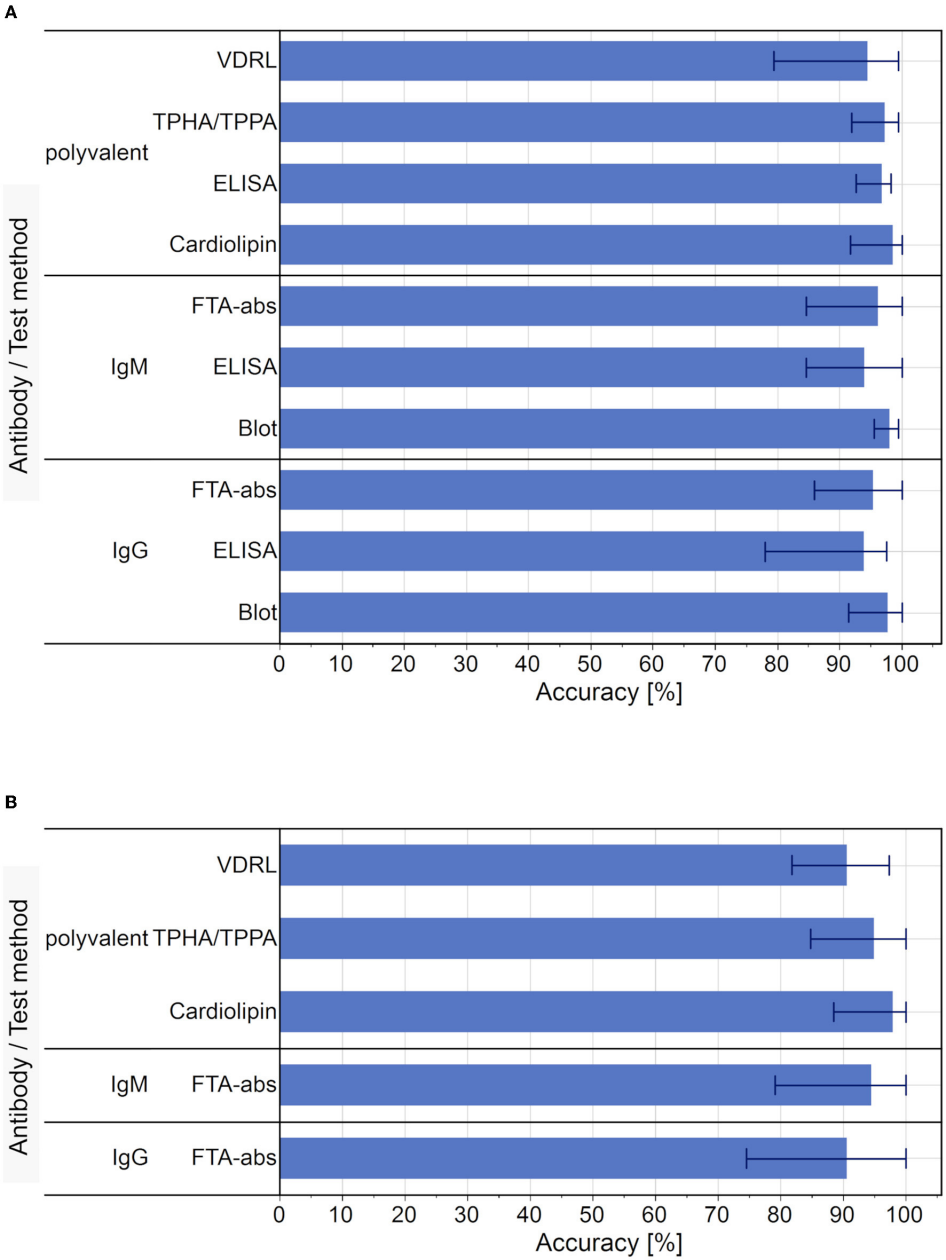
Average accuracy rate of test results for each test used in the EQA surveys, 2010 to 2012. **(A)** Average accuracy of qualitative test results. Bar markers indicate the range of results. **(B)** Average accuracy of quantitative test results. Bar markers indicate the range of results. Blot and ELISA tests were not performed as part of the quantitative diagnostic testing for syphilis.

We analyzed the data from six Europe-wide EQA surveys for *Treponema pallidum* antibodies conducted between 2010 and 2012. In this period, an average of 1,033 laboratory results were reported per year (2010: 892; 2011: 1,191; 2012: 1,014). The mean accuracy rate of the qualitative test results (96.4 %; range 78.0–100 %) was slightly higher than that of the quantitative test results (94.0 %, range 74.5–100 %). The accuracy rate for the different detection methods used in the EQA schemes is displayed in Figure 6.

In terms of the qualitative results, the cardiolipin detection had the highest average accuracy (98.6 %, range 91.7–100 %) and the IgG-ELISA had the lowest (94.0 %, range 78.0–97.6 %). In the case of the quantitative results, Cardiolipin tests also showed the highest average accuracy (98.0 %, range 88.5–100 %) and the IgG-FTA-abs detection methods showed the lowest average accuracy rate (90.6 %, range 74.5–100 %).

All serum samples used in these EQA schemes were derived from single donors with a clear clinical history (20–22), which enables the basic performance parameters for the different test methods to be calculated for the model. The observed average sensitivity for the recommended sequential testing for syphilis via qualitative test methods was 92.8 % and the combined specificity was 99.9 %.

### Assessment of Possible Effect of Test Quality on Estimated Cases

Our estimated incidence rate for syphilis (6.5/100,000 standardized person-years) would result in an estimated 5,256 syphilis cases for the German reference population. In our model, the average sensitivity (92.8 %) and specificity (99.9 %) of sequential screening for syphilis antibodies in the EQA results leads to in 4,880 true positive and 64,195 false positive cases. The specificity 80,798,298 true negative and 377 false negative test results in the German reference population.

These quality parameters of the test algorithm and the estimated average incidence for syphilis in Germany would result in a PPV of 7.1 % and an NPV of > 99.9 % if the test algorithm were used for population-wide screening purposes.

When the estimated seroprevalence based on first-time blood donors (78.2/100,000 standardized person-years) was used to calculate both PPV and NPV for a possible population-based screening for seroprevalence of syphilis antibodies, the PPV would increase to 47.7 % while the NPV would remain at > 99.9 %.

### Economic Evaluation of the Insured Population

Based on the insurance dataset, we calculated the average annual direct and indirect costs for syphilis in our insurance population.

Between 2010 and 2012, an average of 72,234 tests were performed each year (TPHA/TPPA & ELISA: 93 %, VDRL test: 7 %, FTA-abs IgG test & blots: 1 %). As the average cost for testing statutory insured outpatients was €4.96 per test, the average annual costs for screening and confirmatory testing amounted to €358,087.

In the insured population, an average of 312 incident cases of syphilis, without syphilis connata were diagnosed in an outpatient setting. 108 cases were treated as early and 106 as late syphilis; both groups were treated with benzathine penicillin, doxycycline and ceftriaxone in accordance with German treatment guidelines (12). Since the exact treatment regime of the average 98 patients with unspecified syphilis (A53.x) was unknown, they were excluded from this analysis. The average annual per-patient cost of benzathine penicillin, doxycycline or ceftriaxone in outpatients was calculated at €33.24, €9.40 and €184.70 respectively for early syphilis and €99.72, €18.80 and €258.58 respectively for late syphilis. This led to an estimated average annual cost for early and late outpatient treatment of €3,584 and €3,455 respectively. The average annual number of inpatients was 49 patients with an individual median length of stay of 6 days per year (min. 1 day, max. 55 days) average annual cost of €118,503.

236 outpatient and 40 inpatient cases in the age group of 18 to 64 years were included in the calculation of productivity loss on the basis of an average cost of €100.40 per sick day in accordance with the Hanover consent. During 2010 and 2012, inpatients were absent from work for a median of 6 days per year and patient (min. 1 day, max. 446 days), while 4.7 % of all observed outpatients were absent from work for a median of 15 days per year and patient (min. 1 day, max. 546 days), resulting in average annual indirect costs of €24,096 for inpatients and €16,705 for outpatients.

Taken together, the average annual burden for our insurance population was €524,430 for the observed period (Table 3).

**Table 3:** Economic burden of syphilis in the insurance study population. Missing data was coded n.I. (no information).

**Average annual expected costs (**€**)**	
**Direct costs**	
**Treatment, outpatients (*N =* 312)**	
Early (A51.x) (*N =* 108)	3,584
Late (A52.x) (*N =* 106)	3,455
Unspecified (A53.x) (*N =* 98)	n.I.
Total treatment, outpatients	7,039
**Screening and confirmatory testing, outpatients (*N =* 72,234)**	
Total screening and confirmatory testing, outpatients	358,087
**Inpatient care (*N =* 49)**	118,503
**Total direct costs**	***483,630***
**Indirect costs (*N =* 276)**	
Productivity losses^a^, outpatients (*N =* 236)	16,705
Productivity losses^a^, inpatients (*N =* 40)	24,096
**Total indirect costs**	***40,801***
***Total cost of syphilis***	***524,430***

a *Productivity losses are costs due to the incapacity to work*.

### Extrapolated Economic Evaluation of Syphilis in Germany

The expected average number of 5,256 incident cases of syphilis per year is based on the German standard population and our estimated incidence rate of 6.5/100,000 standardized person-years. Using the inpatient-outpatient ratio from our insurance population as a reference, this would result in an average of 4,573 outpatients and 683 inpatients annually when extrapolated to the German standard population. This would produce expected treatment costs for outpatients of €99,960. The treatment of the expected inpatients would lead to an average annual economic burden to healthcare of €1,652,466.

Based on our insurance data, it was estimated that an average of 1,006,727 tests would be performed, leading to a total average annual cost for screening and confirmatory testing of €5,787,886 for outpatients. Since about 10 % of the German population is covered by private health insurance, the total estimated costs for laboratory tests consist of €1,296,279 for privately insured persons and €4,491,607 for persons covered by statutory health insurance. The differences in costs were based on the different fee catalogs of the statutory and private health insurance companies in Germany (27, 28).

Based on average population data, 67,2% of all patients were estimated to be of working age (age group 18 to 64 years), leading to a total of 3,074 expected outpatients and 460 expected inpatients. As for social costs, we estimated the total average indirect costs to be €494,375 annually.

Thus, the total average cost of syphilis for the entire German population is estimated to be €8,034,688 annually during our study period (Table 4).

**Table 4:** Expected economic burden of syphilis in Germany. Missing data was coded n.I. (no information).

**Average annual expected costs (**€**)**	
**Direct costs**	
**Antibiotic treatment, outpatients *N =* 4,573)**	
Early (A51.x) (*N =* 1,534)	50,894
Late (A52.x) (*N =* 1,505)	49,066
Unspecified (A53.x) (*N =* 1,534)	n.I.
Total treatment, outpatients	99,960
**Screening and confirmatory testing, outpatients (*N =* 1,006,727)**	
Statutory insured (*N =* 906,054)	4,491,607
Privately insured (*N =* 100,673)	1,296,279
Total screening and confirmatory testing, outpatients	5,787,886
**Inpatient care (*N =* 683)**	1,652,466
**Total direct costs**	***7,540,312***
**Indirect costs (*N =* 3,534)**	
Productivity losses^a^, outpatients (*N =* 3,074)	217,623
Productivity losses^a^, inpatients (*N =* 460)	276,752
**Total indirect costs**	***494,375***
***Total cost of syphilis***	***8,034,688***

a *Productivity losses are costs due to the incapacity to work*.

With respect to the cost of screening and testing in the blood donor population, 2,472,587 tests were performed on blood donors in Germany, leading to a further €12,257,422 in screening and confirmatory testing costs.

Taken together, the cost for blood sample screening as well as the number of estimated costs based on health insurance data in our model would result in an estimated annual cost of €20,292,110.

## Discussion

In this paper, we present a multi-dimensional retrospective model analysis that combines several data sources to estimate the expected burden of syphilis on the German healthcare system. This includes an economic estimation, which we evaluated based on data for the years 2010 to 2012.

Since all syphilis-positive diagnoses must be reported anonymously to the RKI, we have an overview of incident cases in Germany. From 2010 to 2012 the incidence rose from 5/100,000 person-years to 6.2/100,000 persons with an average of 5.7 cases/100,000 inhabitants (17). Meanwhile, the incidence rate in our insurance dataset declined from 7.2/100,000 cases to 5.6/100,000 cases. This contrasting development of incidences was most likely due to the differences in the two populations as well as the composition and number of members of the German statutory healthcare providers (e.g., socio-economic status, gender distribution) (13).

Both populations furthermore differed in age structure and therefore we used the ASR approach to calculate the expected incidence rate for the German population between 2010 and 2012 based on our insurance dataset. The estimated incidence rates were 7.4 [CI: 6.7–8.1] incident cases per 100,000 standardized person-years in 2010, which dropped to 5.9 [CI: 5.2–6.5] incident cases per 100,000 standardized person-years in 2012, with an average of 6.5 [CI: 5.8–7.2] incident cases per 100,00 standardized person-years in this study period.

The higher incidence rate in our insurance cohort in comparison to the reported cases could be an indication of a slight underreporting of syphilis cases in Germany. This could be due to the fact that only positive laboratory results have to be reported (8), not the clinical diagnosis in general, which is why certain data could be missing in the RKI dataset. Furthermore, the RKI data do not include cases of residual titers of past infections, suspected double reporting, or suspected cases of insufficiently treated syphilis (*syphilis non satis curate)* (18). Taken together, a slightly higher incidence within the claims data was to be expected. This highlights the usefulness of the inclusion of health insurance data in these models, since all the excluded cases can also impact the healthcare system–be it further testing or treatment costs in the case of *syphilis non satis curate*.

The unusually high number of syphilis cases among the older female insurance population could have several reasons: it could be an artifact within the dataset, or the age group might consist of a bigger proportion of women at a higher risk of being infected with syphilis, or it could be a remnant of the surviving World War II generation. Since *Treponema pallidum*-specific antibodies show a lifelong persistence regardless of treatment status (30), a rising seroprevalence of *Treponema pallidum*-specific antibodies is not uncommon in aging populations (31–33).

Despite the possible limitations of our dataset, we were able to identify regional hotspots of syphilis infections in the different federal states in Germany in line with the actual reported data. In particular, the higher number of syphilis cases in this region could have been an indicator of a rise in cases since the reported incidence in the federal state of Rhineland-Palatinate rose from 3.8/100,000 person-years to 5.0/100,000 person-years (+ 31.6 %) from 2012 to 2013 (17).

Since the realization that *Treponema pallidum* could be transmitted via blood, screening tests for syphilis are routinely performed (34) and it is still mandatory for a blood donor sample to be negative for *Treponema pallidum* antibodies before it can be released for donation (35). In our study period, the mean seroprevalence for *Treponema pallidum* specific antibodies in first-time blood donors was 42.5/100,000 donations (0.04 % of all samples) and therefore notably higher than the reported incidence rates. These numbers were still considerably low in comparison to other countries, like the USA (36) or less industrialized countries (32, 37, 38). Data from blood donor surveys are a suitable tool for monitoring infectious disease (11, 12) and might reflect the German population better than our insurance cohort alone. We concentrated our model on first-time blood donors since they are less monitored than repeat donors, are often unaware of their behavioral risks (38, 39), and are less likely to be affected by selection bias (36).

After ASR normalization, the seropositive rate rose to an average seroprevalence rate of 78.2 [CI: 61.1-95.4]/100,000 person-years. Since a thorough anamnesis of current and past infections, including syphilis, is mandatory in Germany (35), one could assume that the observed seroprevalence indicates a high factor of underassessment in the German population. But since the anamnesis is mostly done by questionnaires, it is prone to errors or information bias. Other publications have already highlighted the fact that donors sometimes give false or insufficient information about their health history and possible risk factors (40–42). In Germany, a notable number of male seropositive donors stated that their way of potentially becoming infected was having sex with other men (MSM), a group that was originally excluded from blood donations at the time due to their high risk for STIs (12, 29). Therefore, the data might include people from risk groups as well as people who have already recovered from a syphilis infection. As the assessment is made by directly testing for syphilis, which detects lifelong acquired anti-*Treponema* antibodies, it is not possible to distinguish between a cured or an active infection, as discussed above with respect to the older insurance cohort.

We used the data on laboratory diagnosis, therapy and days absent from work from the insurance dataset to estimate the possible direct and indirect economic burden of syphilis for Germany in our study period. The average annual costs in our insurance cohort amounted to €524,430 in our study period. When extrapolated to the corresponding German population, this would amount to an estimated total annual economic burden for the diagnosis and treatment of syphilis of €8,034,688.

Combining this with the costs for screening all blood donor samples (first-time and repeated), the total estimated economic burden of syphilis based on our model would amount to €20,292,110. In comparison to the average annual total healthcare costs from 2010 to 2012 as 29,730 Mio. € (https://www.gbe-bund.de/), the individual contribution of syphilis might be small, but one should keep in mind that the general incidence of syphilis in Germany was quite low at that time. A rising incidence could change the importance of this infectious disease within a few years, so these model analyses are a useful tool to estimate the expected economic burden.

In our study we used data from 2010 to 2012, because these data were available for our research. Notably, the average number of reported cases from RKI in the period from 2013 to 2019 was almost doubled in comparison with the average number of reported cases in the period from 2010 to 2012, with a peak of 7880 cases in 2019. This 1.5-fold increase in cases would result in €2.6 treatment costs for in- and outpatients. In addition the test costs would rise to 8.7 mio. Forthermore, from the societal point of view, we projected the total indirect costs to be 681,285 €. Thus, the total average cost of syphilis for the entire German population is projected to be nearly €12 Mio, without including blood donor testing costs.

To test the extrapolations of our insurance cohort, we compared the number of expected syphilis inpatients with the number of actual reported cases. Despite the limitations of our insurance cohort, the extrapolation of an average of 683 expected inpatients per year underestimated the actual number of patients reported under the DRG by 10.8 % (on average 766 cases per year) (43).

Finally, we combined the extrapolated data from the insurance dataset with the accuracy of the test systems observed in the evaluation of international EQA surveys conducted by INSTAND between 2010 and 2012. The EQA schemes for syphilis antibodies are performed with sample material from single donors with a clear medical history (20–22), which makes bias due to matrix effects unlikely.

The various test systems showed high accuracies for qualitative (96.4 %, range: 78.0 %- 100 %) und quantitative (94.0, range 74.5–100 %) test results, which are slightly higher than previously reported results (44). Using the known diagnosis for the EQA sample as a gold standard, we calculated the average sensitivity and specificity within the observed period to be 92.8 and 99.9 %, respectively.

The use of the reverse test algorithm for *Treponema pallidum* on the basis of the reference population would result in 64,195 false-positive results and 377 false-negative results. While the false-negative results could lead to a further rise in syphilis cases due to transmission, the falsepositive results would result in overtreatment.

Our study has some limitations. First, the insurance dataset differed from the German reference population with respect to age and male-to-female ratio. German statutory healthcare providers differ in terms of composition (e.g., socio-economic status, gender distribution) and number of members (13); therefore, the use of data from just one insurance institution could not be representative enough and data from further insurance providers should be included in future models to strengthen the validity.

We were unable to adjust for possible differences in socio-economic status of both populations because the information was missing in the DAK-G dataset. A low socio-economic status is a risk factor for syphilis infection (31, 36) and an unnoted difference between both datasets could contribute to the observed differences in the estimated and reported incidence rates. We did not include costs derived from possible coinfections, like HIV, even though an active syphilis infection is a known risk factor for the transmission of HIV (45, 46). This impact could be quite notable due to the high costs of anti-retroviral therapy (47), but we wanted to develop a model focusing on syphilis, which should be more easily transferable to other infectious diseases. Furthermore, we were unable to include the impact of short-term absence from work (> 3 days) since this information was missing as well. The limitation of the blood-donor dataset includes a possible underestimation of the seroprevalence based on this exclusion criteria for risk groups (12). Since we were missing detailed information about the treatment regime for outpatients coded with unspecified syphilis (A53.x), we could not include them in our economic evaluation of the direct costs. This leads to a slight underestimation of direct treatment costs for outpatients.

The strength of our model concerning the linkage of real-life population data, based on claims data from a large statutory health insurance company in Germany, with surveillance data from blood donors and the observed test quality based on international EQA schemes. Futhermore, our results show a good condordance with corresponding DRG data.

These results show the usefulness of claims data in estimating the (economic) burden of a disease on the corresponding healthcare system, especially since they often include data other than health surveys, including most prominently a treatment algorithm. Our model was able to predict the expected number of syphilis inpatients with an accuracy of 89.2 %. The assessment of the ‘real-life' test quality, as estimated based on our EQA schemes, is a useful tool to further estimate the possible impact of overtreatment and possible underestimation based on test quality.

## Conclusions

The linkage of claims data, results from EQA schemes and screening information, such as blood donor surveillance, can be a useful tool for assessing the burden of certain diseases like syphilis on the healthcare system–including the financial burden and indirect economic burden due to absence from work. This model might help to raise the awareness of health care professionals in special geographic regions.

Furthermore, it can help to raise awareness in possible at-risk populations and may help to estimate the minimum future budgets. The use of old datasets is useful to test the strengths and weaknesses of such model analysis.

## Data Availability Statement

The original contributions presented in the study are included in the article/Supplementary Material, further inquiries can be directed to the corresponding author.

## Ethics Statement

The studies involving human participants were reviewed and approved by Ethics Committee of Goethe University Frankfurt (Main), which approved the usage of samples from voluntary blood donors to be used for the EQA schemes. Written informed consent for participation was not required for this study in accordance with the national legislation and the institutional requirements.

## Author Contributions

RŠ, FN, BL, and K-PH contributed to conception and design of the study. RS, NW, LV, and BL performed the statistical analysis. RS and NW wrote the first draft of the manuscript. RŠ and NW share the first authorship. KPH and BL contributed equally to this work and share the last authorship. All authors contributed to manuscript revision, read and approved the submitted version.

## Funding

The research was performed as part of an employment of the authors and by the INSTAND grant VF-01-KPH-2017.

## Conflict of Interest

The authors declare that the research was conducted in the absence of any commercial or financial relationships that could be construed as a potential conflict of interest.

## Publisher's Note

All claims expressed in this article are solely those of the authors and do not necessarily represent those of their affiliated organizations, or those of the publisher, the editors and the reviewers. Any product that may be evaluated in this article, or claim that may be made by its manufacturer, is not guaranteed or endorsed by the publisher.

## Abbreviations

ASR: age standardized rates
DAK-G: Deutsche Angestellten Krankenkasse-Gesundheit
DRG: diagnosis-related group
EBM: Einheitlicher Bewertungsmassstab
EQA: external quality assessment
GoAe: Gebuehrenordnung fuer Aerzte
RKI: Robert Koch Institute.
